# Transmission Characteristics of Primate Vocalizations: Implications for Acoustic Analyses

**DOI:** 10.1371/journal.pone.0023015

**Published:** 2011-08-01

**Authors:** Peter Maciej, Julia Fischer, Kurt Hammerschmidt

**Affiliations:** Cognitive Ethology Laboratory, German Primate Center, Göttingen, Germany; University of Sussex, United Kingdom

## Abstract

Acoustic analyses have become a staple method in field studies of animal vocal communication, with nearly all investigations using computer-based approaches to extract specific features from sounds. Various algorithms can be used to extract acoustic variables that may then be related to variables such as individual identity, context or reproductive state. Habitat structure and recording conditions, however, have strong effects on the acoustic structure of sound signals. The purpose of this study was to identify which acoustic parameters reliably describe features of propagated sounds. We conducted broadcast experiments and examined the influence of habitat type, transmission height, and re-recording distance on the validity (deviation from the original sound) and reliability (variation within identical recording conditions) of acoustic features of different primate call types. Validity and reliability varied independently of each other in relation to habitat, transmission height, and re-recording distance, and depended strongly on the call type. The smallest deviations from the original sounds were obtained by a visually-controlled calculation of the fundamental frequency. Start- and end parameters of a sound were most susceptible to degradation in the environment. Because the recording conditions can have appreciable effects on acoustic parameters, it is advisable to validate the extraction method of acoustic variables from recordings over longer distances before using them in acoustic analyses.

## Introduction

With the advent of affordable recording equipment and computer-based analytical tools, acoustic analyses have become an important part of ethological research. Formal training in bioacoustics is frequently lacking from standard curricula, and despite the existence of some first rate textbooks on the subject, such as Bradbury and Vehrencamp's Principles of Animal Communication [1], most novices are faced with sketchy information regarding methodological pitfalls and considerations. Particularly when it comes to the analysis of vocalizations recorded in the field, a number of problems may arise when measurements are taken from spectrograms or amplitude waveforms. Specially, recording distance, calling height and habitat structure may have a strong effect on different acoustic variables.

Many of the earlier bioacoustic studies, as well as the majority of current studies of bird song [2]–[4] were based on the visual classification of sound spectrograms. Studies on insects and anuran acoustic communication, in contrast, frequently relied on call amplitude and temporal patterns [5]–[7]. Studies of more complex sounds, such as bird calls [8], [9], carnivore vocalizations [10]–[12] and primate calls [13]–[16] applied various algorithms to extract different features from the frequency-time matrix (spectrum) determined by the means of the Fourier transform (for description see [1]).

In particular, in studies of mammalian vocalizations a detailed description of energy distribution can be useful to describe differences related to sender variables such as individual identity, context or affective state. Some commercially available or public domain sound analysis programs (e.g. Avisoft SASLab (R. Specht, Berlin), RAVEN (Cornell Lab of Ornithology), PRAAT (Institute of Phonetic Science, http://www.praat.org) or Signal (Engeneering Design, Belmont, MA)) may offer the calculation of acoustic variables describing various acoustic features, while other studies make use of custom software programs to determine different sets of acoustic features [17]–[19]. Depending on the type of the program and the vocalizations under study, such software programs may determine the location and modulation of the fundamental frequency, the statistical distribution of the amplitude in the frequency spectrum, the peak frequency, and so on. In recent years, several studies applied LPC analyses (linear predictive coding [20]) to extract formants from animal vocalizations [21]–[25]. Such analyses yield measurements such as the location and width of the formants in the frequency spectrum.

The purpose of this study was to assess which acoustic parameters are particularly susceptible to degradation during sound propagation. It is well known that propagation distance has frequency dependent effects on sound transmission [26]–[29]. In addition, numerous studies have demonstrated that different habitats vary in terms of reflections, scattering of sound, and background noise, which all lead to additional differences in signal attenuation and reverberation [1], [28], [30]–[36].

In this study we examined the influence of habitat type, transmission height and re-recording distance on the variation of several acoustic features when rerecorded under different conditions. As examples, we broadcasted and rerecorded a set of calls that we recorded from baboons (*Papio* spp.). We then assessed the effect of the different recording conditions on the reliability and validity of the parameter determination, using the custom software program LMA 2010 as an exemplary tool for the calculation of acoustic features. Based on these findings, we discuss the aspects which should be taken into account when field recordings are analyzed.

## Methods

### Ethics Statement

The paper is based on playback experiments conducted in Germany in which calls were used that had been recorded as part of a series of studies in African National Parks. For each study, permission was granted by the respective local authorities to the head researcher(s) of the field projects. Recordings from baboons in the Moremi Wildlife reserve were made by permission from the Office of the President and the Department of Wildlife and National Parks of the Republic of Botswana to Robert M. Seyfarth and Dorothy L. Cheney (JF was a postdoctoral fellow of theirs and made the recordings between 1997–1999). Recordings in Tsaobis Leopard Park were made by Kristine Meise under research permission from the Ministry of Lands and Resettlement (2006– 2007) and the Ministry of Environment and Tourism to Guy Cowlishaw.

### Recording Experiments

We conducted transmission experiments in the Nature Park ‘Kerslingeroeder Feld’ in the Goettinger Forest, Germany. The ‘Kerslingeroeder Feld’ is a 200 ha neglected grassland with high structured forest edges and old beech woodland. The grassland is characterized by open hay meadows and pastures. The beech woodland consists of deciduous forest with little undergrowth including mainly beech (*Fagus sylvatica*), oak (*Quercus robur*) and alder (*Acer* spp.).

**Figure 1:**
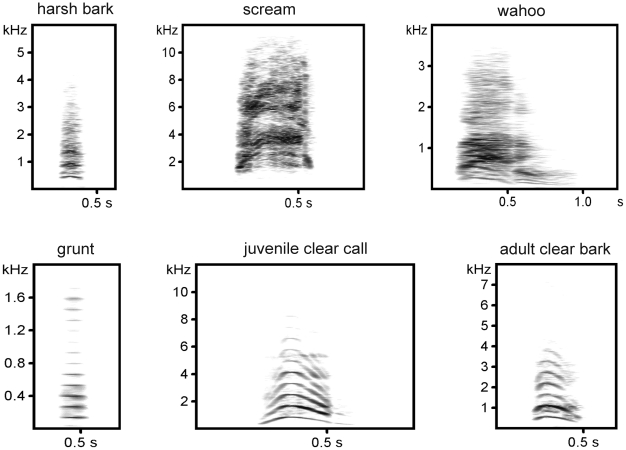
Broadcasted call types. The sampling frequency was adjusted for each call type.

The audio recordings were recorded from Chacma baboons (*Papio cynocephalus*) living in the Moremi Wildlife Reserve, Botswana [14], [23] and in the Tsaobis Leopard Park, Namibia [37]. To assess the variation in relation to differences in call structure, we chose six call types that represent the spectrum of baboon vocal repertoire: ‘harsh barks’, ‘screams’, ‘wahoos’, ‘grunts’, ‘clear calls’ and ‘clear barks’. ‘Harsh barks’ are given by adult baboons in response to large predators [23], [38]. ‘Screams’ are very loud, harsh calls that are given by any individual mostly during aggressive interactions [39]. The two-syllable bark variants or ‘wahoos’ are mainly used by adult male baboons as display calls of male competitive ability or as alarm vocalizations [14], [40]. The soft modulated ‘grunts’ are the most common short-distance baboon vocalizations [41], are harmonically rich, and occur in a variety of social and non-social contexts [37], [42]. The juvenile ‘clear calls’ and the adult ‘clear barks’ are harmonically rich loud calls given when at risk of losing contact with the group or when separated from particular individuals [43]–[45]. Figure 1 presents spectrograms of the different call types. To take the inter-individual variability into account, we used calls from five different individuals for each call type. The recording distances varied between call types: harsh barks were recorded from a distance of 8–12 m; screams at 3–5 m, wahoos at 8–12 m, grunts at 2–3 m, juvenile clear calls 3–5 m, and clear barks at 8–10 m. Note that information on recording distance was only available for bouts, but not for individual calls. Because the recording distance was constant within the denoted range for each call type (see above), the variable ‘original recording distance’ was not entered into the analysis. For the same reason, it is not possible to differentiate between the variation explained by the structure of the call and the one explained by variation in original recording distance. As a first pass at this question, we did an additional calculation with calls recorded below 5 m only (screams, grunts, and juvenile clear calls). In this analysis, the recording distance was below the re-recording distance, thus minimizing potential effects of signal degradation between the calling animal and the microphone.

As recording equipment, we used SonyWMTCD-100 DAT recorder or Marantz PMD660 solid-state recorder with Sennheiser directional microphone with a K6 power module and ME66 recording head and a MZW66 pro windscreen.

For the transmission experiments we varied the following factors: 1) habitat: open field or deciduous forest; 2) transmission height, i.e. loudspeaker and microphone were set at the same height of 0.5 m or 2 m above the ground; 3) distance between sound source and microphone: 6.25 m, 12.5 m, 25 m and 50 m. Distances and heights were measured using a measuring tape (length 50 m). In both habitats, we broadcasted and rerecorded the sounds ten times from both heights and each distance. To test to which extent the results of the experiment can be validly generalized to differing habitat conditions; we additionally broadcasted the same sounds once at five different locations (four locations together with one repetition from the locality of the first experiment) in both habitats and varied the other conditions as in the former experiment. In total we broadcasted and analyzed 6720 calls.

Calls were played back using an active speaker (David Active, VISONIK, Berlin) connected to a Marantz PMD-660 recorder. We rerecorded sounds using a Marantz PMD-661 SD-card recorder (48 kHz sampling frequency, 16 bit) and a Sennheiser directional microphone (K6 power module and ME66 recording head with ME66 Rycote windscreen). The active speaker and microphone were fixed on tripods. We measured the sound pressure level (dB) of the broadcasted calls by using a VOLTCRAFT 322 sound level meter (settings: ‘C’ weighting, response time: 125 ms). Table 1 shows the dB values of each call broadcasted in a sound proof chamber from 1.5 m distance.

**Table 1:** Sound pressure level (dB) of broadcasted calls measured at 1.5 m distance from the loudspeaker in a sound proof chamber.

Individual	harsh bark	scream	wahoo	grunt	clear call	clear bark
1	84.2	93.7	90.1	70.9	95.4	96.9
2	90.0	91.1	92.4	76.6	93.6	96.1
3	83.3	86.5	94.9	79.1	88.0	94.1
4	90.2	83.3	94.9	71.4	88.7	97.3
5	84.2	93.4	93.2	67.9	95.1	91.4

Temperature, humidity and wind speed were measured at each distance. In the deciduous forest the density was measured by using a wooden frame (0.5 m×0.5 m) divided into 100 open wire mesh squares. Measurement consists of a count of the number of squares which are visually obstructed by the vegetation (see [46] for a detailed description). In the open habitat we measured the grass height by using the direct measurement method [47]. The temperature in both habitats ranged between 19 and 24°C and the humidity varied less than 20 %. The density in the forest habitat varied between 30 and 40 obstructed squares and the grass height in the open habitat ranged from 20 to 30 cm. Sounds were only broadcasted when the wind speed was below 3 km/h (anemometer: Siltknecht, Gossau Switzerland). A detailed description of the ecological data is given in Table S1 in the supplementary material.

### Acoustic analyses

To describe the amplitude attenuation over distance and different broadcasting conditions, we calculated the maximal amplitude of the amplitude envelope for each call, using the Program Signal 5.0 (Engineering Design, Belmont, MA).

All broadcasted sounds were recorded with the same equipment settings and recording level was not changed during the experiments. Since we controlled for ambient noise we could automatise the extraction of the sound files, from the records, using the label function of AVISOFT SASLAB Pro (R. Specht, Berlin). To standardize the cutting process we defined a label threshold of 5 % and a fixed margin time of 0.6 s (which means that every waveform event exceeding 5 % of the ambient noise level was labeled and cut with a margin time of 0.6 s at both sides of the call). To obtain an appropriate range for the estimation of the acoustic features of the rerecorded calls we reduced the sampling frequency for each call type: harsh bark  = 16 kHz, scream  = 24 kHz, wahoo  = 16 kHz, grunt  = 4 kHz, clear call  = 16 kHz and clear bark  = 12 kHz. We submitted the resulting frequency time spectra to a custom software program that extracts different sets of parameters from acoustic signals (LMA 2010). To reduce the background noise we set the cut-off frequency at 100 Hz (the frequency range of all calls was above 100 Hz). The start and end thresholds were set at 20 %, which means that all time segments with a value lower than 20 % of the maximal amplitude at the beginning and end of the call were not considered.

Below, we briefly describe the underlying principle for the different groups of measurements. First, we measured the statistical distribution of the frequency amplitudes in the spectrogram (DFA). For each time segment, the overall amplitude was determined. Subsequently, we calculated the frequency at which the distribution of the amplitude reaches the first quartile of the total distribution, respectively (DFA1). Second, we calculated parameters describing the first dominant frequency band (DFB1). The dominant frequency bands are characterized by amplitudes that exceed a given threshold in a consecutive number of frequency bins. The numbers of the dominant frequency bands count from the lowest frequency up; the first DFB is not necessarily the DFB with the highest amplitude. Third, we specified the location of the peak frequency: the frequency with the highest amplitude in a certain time segment (PF). These parameters were extracted by using the general automatic extraction method of LMA.

For the tonal calls we calculated the fundamental frequency (F0), which is the lowest frequency band in harmonic calls. The F0 was calculated by using the tonal macro of LMA which is based on an autocorrelation function. Via this function, only tonal elements of a call are used to calculate the parameter whereas noisy elements are ignored. For the calculation we applied a manual as well as an automated method and compared both results. In both cases the tonality of a time segment was estimated by a cross-correlation algorithm. In the manual macro the possible F0 range is set by visual adjustment of a harmonic curser. The F0 itself was estimated by an algorithm searching the highest frequency amplitude within the range of the lowest cursor. In the automatic macro instead, the F0 is calculated automatically, with an algorithm estimating the least common divisor of the peaks of cross-correlation function. Table 2 provides a detailed description of the acoustic parameters.

**Table 2:** Description of the acoustic parameters used in the analyses.

Parameter	Description
Duration (ms)^1^	duration of the call
DFA 1 mean (Hz)^1^	frequency at which the distribution of frequency amplitudes reaches the first quartile, mean across time segments
DFB1 start (Hz)^2^	first dominant frequency band, at the beginning of the call
DFB1 end (Hz)^2^	first dominant frequency band, at the end of the call
DFB1 mean (Hz)^1^	mean first dominant frequency band across all time segments
PF max (Hz)^1^	frequency of the maximum frequency of the peak frequency across time segments
PF mean (Hz)^1^	mean of the frequencies with the highest amplitude across all time segments
F0 mean (Hz) 3	mean fundamental frequency across all time segments

1 Parameter used for reliability and validity calculation.

2 Parameter only used for reliability calculation.

3 Parameter only used for tonal calls and tonal call parameter calculation.

### Statistical analyses

In principle, there are two ways to explore the quality of the measurements: one is to examine the deviance from the original value (validity), the second is to assess whether a certain call yields the same readings under identical conditions (reliability). To assess the reliability we calculated the coefficient of variation (CV) for each call (n = 10 repetitions per call) under each condition and calculated the mean CV across all calls. To examine the validity, we calculated the differences in percentage between the calls rerecorded in the sound proof chamber ( =  Reference call) and the rerecorded calls at the respective distances and conditions. To compare the influence of the different factors we applied a linear mixed model analysis (SPSS 18.0) with call type, call variant, locality, habitat, re-recording distance and height as fixed factors.

To calculate the accuracy of the fundamental frequency we only analyzed tonal calls and applied again a linear mixed model to examine the influence of the different broadcasting conditions. To compare the accuracy of different extraction methods (manual vs. automatic) we visually compared the resulting parameters.

## Results

### Amplitude attenuation

**Figure 2:**
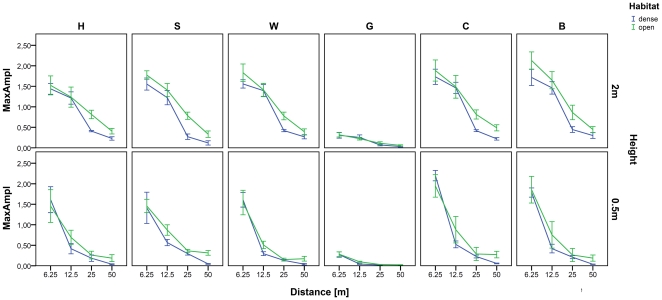
Amplitude attenuation in relation to habitat type, distances and calling height. The call types are shown in the different panels: H =  harsh bark, S =  scream, W =  wahoo, G =  grunt, C =  clear call, B =  clear bark. The maximal amplitude of the amplitude envelope (MaxAmpl) is calculated in mV. Mean values and standard errors are indicated.

Over longer distances signals showed stronger attenuation for both habitat types and transmission heights. At every distance (except at 0.5 m height and 6.25 m distance) the attenuation was stronger for the forest condition compared to the open field condition. Figure 2 shows the mean values for each call type. Under both habitat conditions, the signal attenuation was much stronger at low compared to the higher transmission height. For all call types at low transmission height the maximal amplitude decreased strongly already at a distance of 12.5 m. For calls broadcast in the dense habitat at low broadcasting height, amplitudes of the calls were reliably recordable (calculable) only until 25 m. Grunts were reliably recordable only until 6.25 m at low broadcasting height in both habitats. In general, signal attenuation was strongest at dense field conditions and low transmission height, and lowest in open field conditions and high transmission height.

### Call structure

#### Reliability

Table 3 shows the mean CV values for each call type and parameter. The acoustic parameters describing the course of the first dominant frequency band (DFB1 start and DFB1 end) resulted in a large variability (>10 %) for each call type and hence a poor reliability. The other acoustic parameters yielded a mean variability of less than 5 % (except Pf max  = 5.27 %). The fundamental frequency (F0) in tonal calls and the DFA parameter (DFA 1mean) yielded the largest accuracy and showed variation of less than 3 %. Grunts showed the largest variability compared to the other call types. It is the only call type that showed a variation of more than 20 % for two general parameters (DFB 1 start and DFB 1end).

**Table 3:** Reliability in relation to call type and acoustic parameters measured as coefficient of variation (CV).

Call	Duration (ms)	DFA 1mean (Hz)	DFB 1start (Hz)	DFB 1end (Hz)	DFB 1mean (Hz)	Pf max (Hz)	Pf mean (Hz)	F0 (Hz)
harsh bark	1.44 %	1.34 %	10.07 %	15.00 %	4.38 %	3.48 %	3.16 %	-
scream	1.83 %	1.58 %	15.54 %	17.14 %	4.20 %	3.32 %	2.03 %	-
wahoo	4.30 %	1.67 %	12.30 %	17.19 %	4.39 %	4.99 %	3.44 %	-
grunt	9.20 %	4.45 %	27.46 %	24.97 %	6.47 %	12.36 %	8.34 %	2.20 %
clear call	1.36 %	1.34 %	14.58 %	14.31 %	3.08 %	2.37 %	1.66 %	4.30 %
clear bark	3.00 %	1.52 %	11.77 %	18.09 %	2.93 %	5.11 %	3.32 %	1.67 %
**Mean**	**3.52 %**	**1.99 %**	**15.29 %**	**17.78 %**	**4.24 %**	**5.27 %**	**3.66 %**	**2.73 %**

The CV-values represent mean values across the different conditions for each call, broadcasted and rerecorded ten times at each condition.

#### Validity

**Figure 3:**
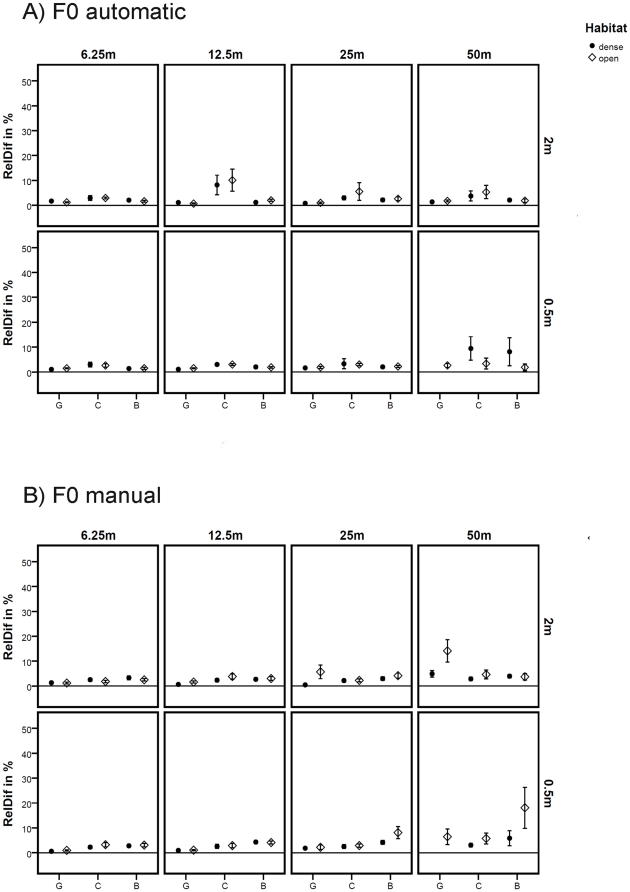
Variability of the fundamental frequency (F0) by using two different calculation methods. The x-axis shows the different call types, G: grunt, C: clear call and B: clear bark. The y-axis shows the relative differences of the acoustic parameters for each condition. Panel rows represent different heights, while panel columns represent different distances. (A) The variability under different conditions calculated via the automatic macro. (B) The variability calculated manually. The plots indicate mean values from ten repetitions at one locality; error bars indicate the confidence interval of 95 %. The horizontal lines denote a variation of 0 %.

The F0 parameter revealed a high accuracy in the automatic tonal extraction method; there were no significant differences in the measurements between the reference calls and the rerecorded calls under different conditions (Table 4). The two methods (automatic vs. manual) for extracting the F0 yielded similar results. Both methods revealed a high accuracy, with some advantage for the manual determination for specific calls and under specific circumstances (Figure 3).

**Table 4:** Validity of the fundamental frequency in tonal calls in relation to distance, height and habitat.

Tonal calls	Call variant	Locality	Distance	Height	Habitat
grunt	2.1	1.8	1.7	1.2	1.5
clear call	5.4*	2.0	2.5	0.8	0.5
clear bark	3.4*	1.3	2.5	0.6	0.8

The table shows F values of the linear mixed model analysis. Values for one repetition of every call at each locality under each condition are calculated. Grunts at 50 m distance were excluded from the analysis.

*p<0.05.

**Figure 4:**
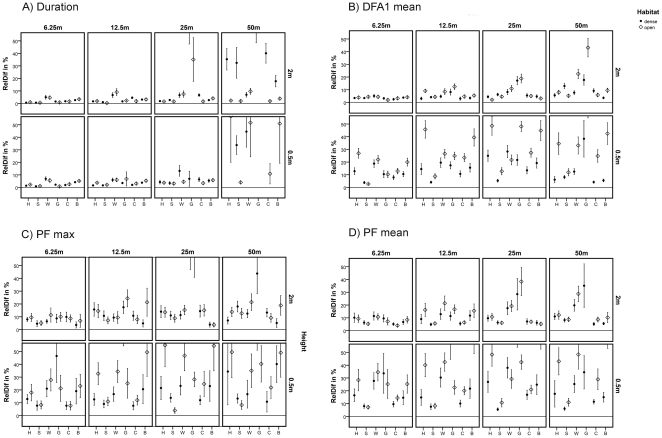
Validity of four different parameters in relation to habitat type, transmission height, distances and call type. Four different acoustic parameters are shown in the graphs; (A) Duration, (B) DFA1 mean, (C) PF max, (D) PF mean. The x-axis shows the different call types, H: harsh bark, S: scream, W: wahoo, G: Grunt, C: clear call and B: clear bark. The y-axis shows the relative differences of the acoustic parameters for each condition. Panel rows represent different heights, while panel columns represent different distances. The plots indicate mean values across five different localities; error bars indicate the confidence interval of 95 %. The horizontal lines denote a variation of 0 %.

**Figure 5:**
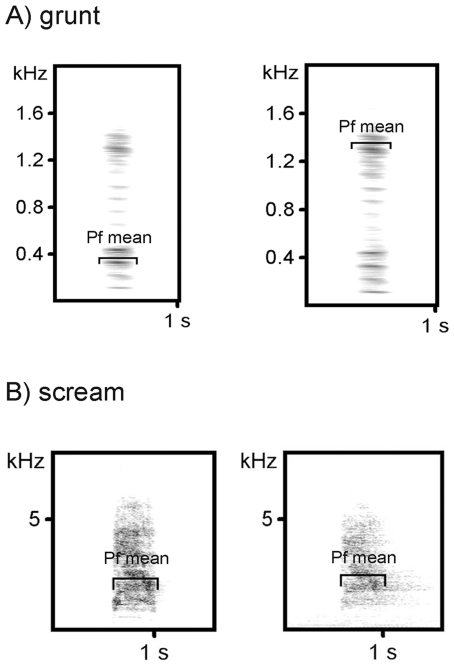
Comparison of peak frequency (PF mean) from two different call types. The Peak frequency (PF mean) of a tonal grunt (A) and a scream (B) recorded in the soundproofed chamber compared to the same grunt recorded in a dense habitat at 0.5 m height and 6.25 m distance and the same scream recorded in a dense habitat at 0.5 m height and 50 m distance. PF mean is indicated.

General call parameter calculation revealed highly significant F values for each call parameter under almost every condition, except for different locations (Table 5). Hence, the calculation differences were strongly influenced by the varying broadcasting conditions. The factor ‘height’ yielded the largest variation for almost every acoustic parameter followed by re-recording distance, call type, habitat and call variant. Different localities in contrast mainly resulted in non-significant differences. Duration was the acoustic parameter with the highest accuracy between the various conditions. Except for long distances, it showed relatively high accuracy for every condition (Figure 4A). By contrast, the distribution of frequency amplitudes (DFA1 mean) only revealed small calculation accuracy. In particular, DFA1 mean was strongly influenced by transmission height (Figure 4B). Transmission height also had a large effect on peak frequency (PF max, PF mean) and in this case caused strong parameter degradation as well (Figure 4C and 4D). Because of the lack of reliability we did not analyze the parameters DFB1 start and DFB1 end. Similar to the reliability calculation, grunts showed the largest differences in the measurements between original calls and rerecorded calls. The spectrogram in Figure 5A shows a grunt example rerecorded in the sound proof chamber and in a dense habitat at 6.25 m distance and 0.5 m height. Screams instead only yielded small differences throughout all different conditions for most of the general acoustic parameters (see Figure 5B for a spectrogram of a scream recorded in a soundproof chamber and at 50 m distance in a dense habitat at 0.5 m height). Table 5 shows the F values of the applied linear mixed model analysis for all the different call parameters under the different conditions. The F-values for each call type are shown separately in the supplementary material, Table S2 A-F. As mentioned before we were not able to include the distance of the original recordings as a continuous covariate. Therefore, we did a separate calculation for the three call types (screams, grunts, and juvenile clear calls) originally recorded at distances below 5 m (Table 6). We found a clear increase in F-values for the factor ‘call type’, and decrease in F values of all other factors. Overall, however, the effects of the different factors were generally following a similar pattern (Table 6).

**Table 5:** Validity of characteristic sound parameters in relation to distance, height and habitat.

Parameter	Call type	Call variant	Locality	Distance	Height	Habitat
Duration	31.9*	1.3	6.3*	116.84*	26.8*	1.6
DFA1 mean	46.9*	11.81*	3.5	47.3*	504.3*	188.8*
DFB1 mean	14.4*	31.8*	0.7	99.2*	99.5*	0.7
Pf max	48.1*	9.7*	1.9	22.0*	66.8*	7.5
Pf mean	50.2*	11.9*	2.2	15.1*	170.0*	33.3*

The table shows F values of the applied linear mixed model analysis. Values for one repetition of every call at each locality under each condition are calculated.

*p<0.05.

**Table 6:** Validity of characteristic sound parameters in relation to distance, height and habitat for calls (screams, grunts, and juvenile clear calls) types with an original recording distance below five meter.

Parameter	Call type	Call variant	Locality	Distance	Height	Habitat
Duration	48.5*	1.8	3.9*	57.2*	3.1	0.6
DFA1 mean	139.5*	1.8	0.7	67.2*	120.1*	70.4*
DFB1 mean	21.6*	30.5*	0.8	28.7*	29*	0.7
Pf max	104.3*	11.3*	0.6	14.9*	4.2*	1.3
Pf mean	109.6*	2.9*	0.3	13.4*	31.4*	0.9

The table shows F values of the applied linear mixed model analysis. Values for one repetition of every call at each locality under each condition are calculated.

*p<0.05.

## Discussion

As expected, we found significant effects of recording conditions on acoustic features. Along with re-recording distance, recording height had a large impact on the validity of acoustic parameter estimation. Calls broadcasted at low heights (e.g. 0.5 m) showed high structural degradation within short distances. Call structure was a further important explanatory factor for the variation in parameter estimation. As long as some harmonics remained, tonal calls showed a high validity in the estimation of parameters describing fundamental frequency (F0). Because we only have one broad estimate of the distance between animal and microphone for each call type, we were unable to assess the influence of the original recording distance on sound degradation within call type. In principle, the analysis of calls that are already notably degraded may lead to over-estimations (cumulative effects) or under-estimations (the sensitive components are already missing in the original recordings) of the effect of propagation. The comparison between call types originally recorded below 5 m and call types originally recorded at about 10 m showed a similar result as the analysis incorporating all call types, however. The most striking difference was that more variation was explained by the factor ‘call type’. This is due not only to the reduced number of call types, but also the fact that the degradation of sounds on parameter estimation has a significant higher influence on noisy than on tonal calls. In this analysis only two tonal and one noisy call type remained. The consideration of these three call types which were originally recorded at a shorter distance enhanced the contrast between tonal and noisy calls in comparison to the first analysis with six call types. In addition, the explained variance of the factor ‘re-recording distance’ and ‘height’ was reduced. Unfortunately, we cannot directly differentiate between the variation explained by the difference in call structure and that explained by the difference in original recording distance. To empirically address the issue of the combined effects of recording distance and re-recording distance, one would need to conduct a study where the distance between animal and microphone is systematically varied. In the present study, we aimed at reducing the variation within call types by selecting calls with a very good quality only.

One of the critical acoustic parameters is DFA (distribution of frequency amplitudes). These parameters describe the statistical distribution of energy in the whole frequency spectrum. Therefore, it is not surprising that the stronger attenuation of low frequencies at lower broadcasting levels makes it difficult to estimate the correct distribution of frequency energy of the original call. Our results are generally in agreement with other researchers’ descriptions of amplitude and frequency dependent attenuation in relation to broadcast conditions and distances [1], [27], [34], [48]-[50]. The high impact on the attenuation of call amplitude and structural degradation at low heights corresponds to the ‘floor effect’ described by Nelson [33]. This effect influences in particular frequencies below 1 kHz. As a consequence the estimation of acoustic parameters is susceptible when calls are transmitted close to the ground.

Parameters describing the peak frequency (PF) are also strongly influenced by broadcasting conditions. Here call structure is an important factor. Calls with dominant single PF peaks (e.g. ‘scream’) are less influenced by broadcasting conditions than call types with several similar amplitude peaks (e.g. ‘grunt’). In such cases small changes in the amplitude of the PF can lead to incorrect identification of a different amplitude peak as the PF (see Figure 5). A further aspect is the frequency range of the highest amplitude. Grunts with a PFs around 300 Hz showed the strongest degradation at the transmission height of 0.5 m. Because baboons give their grunts mostly from the ground, subtle structural variation cannot be transmitted reliably over larger distances. Not surprisingly, these calls are mainly used for short distance communication. Ey and colleagues [51] showed that olive baboons produce grunts with longer call duration in dense habitats, possibly to counterbalance the worse propagation conditions. At higher transmission heights, both DFA and PF parameters revealed a much higher validity even if they were transmitted in the forest habitats. To a lesser degree, this effect was also found in other call types, including harmonically rich loud calls such as ‘clear barks’ and ‘clear calls’. In relation to mean and maximum values, start and end parameters revealed the lowest validity. One reason is that in most call types, start and end parts have a lower amplitude than the rest of the call. Therefore, degradation and absorption has a higher influence on these parts than on the rest of the vocalisation. In addition, the end of calls is most strongly influenced by reverberation over distance [52].

Tonal calls were less susceptible to sound degradation as long as some harmonics remained in the frequency spectra. Although there are different ways to calculate the F0, many algorithms focus on the estimation of the autocorrelation function of the frequency spectra [18], [53]. The autocorrelation function is able to recalculate the F0 of degraded spectra as long as some harmonic peaks remain. In cases in which the degraded spectra have too little harmonic information or the original tonal call has too few harmonics to make a reliable calculation, a visual control of the F0 proposed by the algorithm can lead to a higher reliability of F0 calculation (see Figure 3). Other sound analysis programs, like Avisoft SASLab, PRAAT or SIGNAL offer the possibility to determine the range of F0. This is an alternative possibility to increase the reliability of F0 estimation. Temporal parameters, like call duration, depend mainly on the attenuation of sound amplitude. In contrast to open habitats, dense forest vegetations can cause considerable reverberation and absorption of a signal [18], [28], [52], [54].

In sum, our results suggest that the estimation of acoustic parameters recorded from larger distances, especially transmitted by callers on the ground, lead to erratic measurements. Hence, it is advisable to assess the reliability and validity of certain parameters before they are used in further statistical analyses. The estimation of F0 seems to be the only acoustic parameter which can be reliably calculated as long as a strong signal conveys sufficient harmonics. For a higher caller position a higher microphone height might be favorable. However, this can only slightly reduce the described effects and not compensate for them. Although it is not always possible in studies of free-ranging animals to assess the exact distance at which the calls are recorded, it seems to be advisable to include as much information as possible on recording distance, to allow for a judgment of the reliability of the acoustic measurements taken.

Whilst this study shows that baboon vocalizations suffer some distortion when recorded at low transmission height and far distances, further research is required to understand the relevance of this finding to species living in different habitats and having other vocal types, with different physiological sound production mechanisms. It is also necessary to take into account that the information encoded in a given call structure needs only to be transmitted over the distance at which the animal typically communicates. Degradation that occurs at distances greater than an individual’s natural communication range would thus be functionally irrelevant. Unfortunately, very little is known about how call distortion affects the perception of calls in nonhuman primates. From birds we know that they are able to extract the distance of the signaler from the degree of signal degradation [55]. A playback study in African elephants showed very nicely the differences between signal detection and derived information. Although the elephants were able to recognize contact calls of family members under optimal condition up to 2.5 km, they usually achieved reliable recognition at distances below 1–1.5 km [56]. The reason could be that the crucial components of social identity are distorted at a distance above 1 km due to background noise or attenuation effects. Such playback studies that test the influence of sound degradation on conspecifics’ responses are also required in nonhuman primates, before we can fully assess the reliability and validity of acoustic field recordings.

## Supporting Information

Table S1 Ecological data measured at each locality. Temperature, humidity and wind speed were measured every 15 min and the mean values for each locality were calculated. Density measurements and grass height were taken at each distance (for density at both heights as well) and the mean values for each locality were calculated. + Values represent mean values of obstructed squares [47].
(DOC)

Table S2 Validity of each call type in relation to distance, height and habitat. Parts A-F show the F values of the linear mixed model analysis for each call type under each condition. * p<0.05.
(DOC)
