# Supplementary material for: Transmission Characteristics of Primate Vocalizations: Implications for Acoustic Analyses
Source: PLoS One. 2011 Aug 1;6(8):e23015. doi: 10.1371/journal.pone.0023015 (PMC3148239; doi:10.1371/journal.pone.0023015)
Supplement: Table S1 — Ecological data measured at each locality. Temperature, humidity and wind speed were measured every 15 min and the mean values for each locality were calculated. Density measurements and grass height were taken at each distance (for density at both heights as well) and the mean values for each locality were calculated. + Values represent mean values of obstructed squares [47]. (DOC) [file pone.0023015.s001.doc]

Table S1. Ecological data measured at each locality.

|  | Locality | Density^+^ | Temperature | Humidity | Wind speed |
| --- | --- | --- | --- | --- | --- |
|  |  |  | (°C) | (%) | (km/h) |
| Dense | 1 | 35.5 | 20.9 | 65.4 | 0.0 |
|  | 2 | 30.3 | 20.9 | 63.5 | 0.3 |
|  | 3 | 40.2 | 21.3 | 66.5 | 0.0 |
|  | 4 | 31.3 | 20.0 | 72.8 | 0.0 |
|  | 5 | 38.5 | 19.4 | 78.9 | 0.0 |
|  |  | Grass height |  |  |  |
|  |  | (cm) |  |  |  |
| Open | 1 | 29.3 | 19.4 | 83.3 | 2.1 |
|  | 2 | 23.5 | 19.1 | 71.8 | 2.9 |
|  | 3 | 22.0 | 20.8 | 65.1 | 0.5 |
|  | 4 | 20.3 | 23.3 | 66.5 | 0.3 |
|  | 5 | 22.5 | 24.2 | 65.9 | 0.0 |
