# Supplementary material for: Transmission Characteristics of Primate Vocalizations: Implications for Acoustic Analyses
Source: PLoS One. 2011 Aug 1;6(8):e23015. doi: 10.1371/journal.pone.0023015 (PMC3148239; doi:10.1371/journal.pone.0023015)
Supplement: Table S2 — Validity of each call type in relation to distance, height and habitat. Parts A-F show the F values of the linear mixed model analysis for each call type under each condition. * p<0.05. (DOC) [file pone.0023015.s002.doc]

Table S2. Validity of each call type in relation to distance, height and habitat.

**A)**

| harsh bark | Call variant | Locality | Distance | Height | Habitat |
| --- | --- | --- | --- | --- | --- |
|  |  |  |  |  |  |
| Duration | 1.6 | 1.9 | 41.1^*^ | 21.6^*^ | 13.8^*^ |
| DFA1 mean | 12.9^*^ | 1.1 | 8.5^*^ | 196.2^*^ | 62.9^*^ |
| DFB1 mean | 30.9^*^ | 0.9 | 60.1^*^ | 111.2^*^ | 2.7 |
| Pf max | 10.6^*^ | 1.9 | 4.2^*^ | 25.6^*^ | 14.2^*^ |
| Pf mean | 51.7^*^ | 1.5 | 1.8 | 62.6^*^ | 32.4^*^ |

B)

| scream | Call variant | Locality | Distance | Height | Habitat |
| --- | --- | --- | --- | --- | --- |
|  |  |  |  |  |  |
| Duration | 3.3 | 1.1 | 42.9* | 1.0 | 28.1* |
| DFA1 mean | 7.2* | 0.3 | 20.5* | 5.6 | 1.6 |
| DFB1 mean | 114.7* | 0.2 | 15.3* | 67.8* | 0.1 |
| Pf max | 4.1* | 0.3 | 4.5 | 0.2 | 8.0* |
| Pf mean | 18.2* | 0.2 | 1.5 | 7.1* | 5.1 |

C)

| wahoo | Call variant | Locality | Distance | Height | Habitat |
| --- | --- | --- | --- | --- | --- |
|  |  |  |  |  |  |
| Duration | 3.2 | 1.7 | 15.6^*^ | 15.2^*^ | 0.1 |
| DFA1 mean | 43.5^*^ | 3.9 | 8.3^*^ | 172.4^*^ | 24.3^*^ |
| DFB1 mean | 13.6^*^ | 1.2 | 68.2^*^ | 6.2^*^ | 8.6^*^ |
| Pf max | 8.6^*^ | 1.1 | 2.5 | 38.3^*^ | 9.8^*^ |
| Pf mean | 81.9^*^ | 2.5 | 5.1^*^ | 85.1^*^ | 6.1^*^ |

D)

| grunt | Call variant | Locality | Distance | Height | Habitat |
| --- | --- | --- | --- | --- | --- |
|  |  |  |  |  |  |
| Duration | 2.2 | 3.4^*^ | 37.5^*^ | 2.9 | 6.9^*^ |
| DFA1 mean | 1.4 | 0.3 | 74.6^*^ | 65.5^*^ | 34.7^*^ |
| DFB1 mean | 9.7^*^ | 2.6 | 20.0^*^ | 13.7^*^ | 13.1^*^ |
| Pf max | 13.0^*^ | 1.0 | 13.5^*^ | 5.8 | 2.2 |
| Pf mean | 5.4^*^ | 0.6 | 13.7^*^ | 15.2^*^ | 0.7 |

E)

| clear call | Call variant | Locality | Distance | Height | Habitat |
| --- | --- | --- | --- | --- | --- |
|  |  |  |  |  |  |
| Duration | 0.7 | 3.8^*^ | 61.9^*^ | 11.3^*^ | 63.2^*^ |
| DFA1 mean | 15.1^*^ | 0.8 | 8.8^*^ | 140.2^*^ | 51.5^*^ |
| DFB1 mean | 24.6^*^ | 1.1 | 36.3^*^ | 309.3^*^ | 27.4^*^ |
| Pf max | 9.6^*^ | 0.9 | 5.1^*^ | 2.2 | 1.1 |
| Pf mean | 14.0^*^ | 1.5 | 3.0 | 81.7^*^ | 15.7^*^ |

F)

| clear bark | Call variant | Locality | Distance | Height | Habitat |
| --- | --- | --- | --- | --- | --- |
|  |  |  |  |  |  |
| Duration | 1.0 | 1.9 | 37.5^*^ | 30.1^*^ | 16.9^*^ |
| DFA1 mean | 15.8^*^ | 2.2 | 7.7^*^ | 182.8^*^ | 69.2^*^ |
| DFB1 mean | 37.8^*^ | 1.5 | 90.1^*^ | 171.4^*^ | 1.1 |
| Pf max | 37.5^*^ | 2.0 | 4.9^*^ | 73.9^*^ | 13.7^*^ |
| Pf mean | 29.7^*^ | 1.9 | 7.5^*^ | 116.6^*^ | 46.2^*^ |
